# Carbetocin vs. Syntometrine in Prevention of Postpartum Hemorrhage: a Double Blind Randomized Control Trial

**DOI:** 10.5812/ircmj.7881

**Published:** 2013-09-05

**Authors:** Mansoureh Samimi, Azam Imani-Harsini, Masoumeh Abedzadeh-Kalahroudi

**Affiliations:** 1 Department of Obstetrics and Gynecology, Kashan University of Medical Sciences, Kashan, IR Iran; 2 Obstetrician and Gynecologist, Valieasr Hospital, Brojen, IR Iran; 3 Trauma Research Center, Kashan University of Medical Sciences, Kashan, IR Iran

**Keywords:** Carbetocin, Postpartum Hemorrhage, Prevention and Control, Syntometrine

## Abstract

**Background:**

Postpartum hemorrhage is a signiﬁcant cause of maternal mortality and morbidity, worldwide.

**Objectives:**

The aim of this study was to compare the efficacy between carbetocin and syntometrine in prevention of postpartum hemorrhage.

**Materials and Methods:**

This study was a double blind randomized clinical trial that carried out on 200 pregnant women referred to Shabiehkhani maternity center of Kashan, during 2011. The first group received intramuscular syntometrine and the second group received intramuscular carbetocin after placental delivery. All of the participants were followed for 24 hours and blood pressure, pulse rate, uterine tone, hemoglobin concentration at first and 24 hours after delivery, and the need for additional uterotonic drugs and drug side effects were evaluated. Finally all data were analyzed using t-test, chi square tests and logistic regression.

**Results:**

The mean fall in hemoglobin level in the carbetocin group was significantly lower than the syntometrine group (P < 0.001). Also there were significant differences between the two groups, regarding additional uterotonic drug requirements (P = 0.002). Moreover systolic blood pressure and uterine tone immediately and 30 minutes after drug administration were significantly different (P < 0.001). Incidence rate of tachycardia in the carbetocin group was 13%, in contrast to 5% in the syntometrine group (P = 0.04).

**Conclusions:**

This study revealed that carbetocin is more effective than syntometrine in prevention of postpartum hemorrhages. Thus it can be used as a good alternative of syntometrine for low-risk women.

## 1. Introduction

Postpartum hemorrhage (PPH) is a life threatening situation and one of the important causes of maternal mortality and morbidity, worldwide (1). Annually, 14 million women suffer from postpartum hemorrhage, of which about 140,000 individuals die and 1.6 million encounter anemia and its long term problems (2). Postpartum hemorrhage is seen in 18% of deliveries (3). Postpartum hemorrhage prevention is very important. Several studies have shown that the use of uterotonic agents after placental separation can reduce the incidence of PPH by up to 30% - 40% and use of these drugs for PPH prevention is approved by all researchers (4).

The first uterotonic drug was ergometrine that introduces in 1950, while oxytocin and syntometrine were released during 1953 and 1963, respectively. Syntometrine is a combination of 5 IU oxytocin and 0.5 mg ergotamine in every 1 ml (5, 6). This mixture is one of the most common uterotonic drugs that is used during the third stage of labour, because this drug has the rapid onset of action of oxytocin and the continuous effect of ergometrine. Previous studies have reported that the effects of intramuscular (IM) syntometrine are similar with intravenous (IV) oxytocin (7). Ergotamine may increase blood pressure (5) and coronary artery spasm (6), as it can cause vasoconstriction and muscle contraction; therefore in women with hypertension, asthma or cardiac disease, syntometrine is contraindicated.

On the other hand, carbetocin was introduced during 1987 (8). Carbetocin is a long- acting synthetic analogue of oxytocin with a half-life of 40 minutes and 80% bioavailability in IM injection. After IM or IV administration of this drug, uterine contractions start in less than 2 minutes (9). Drug dosage is 100 microgram during the third stage of labour (10).

The common side effects of carbetocin are nausea, vomiting, abdominal pain, hypotension, headache, chilling and pyrexia. Its contraindications are uterine, vaginal or cervical rupture (8).

Carbetocin makes a longer uterine response compared with oxytocin in terms of frequency and amplitude of contractions (11). Moreover, in comparison with oxytocin or syntometrine, it seems to have fewer gastrointestinal and cardiovascular side effects (12).

Several studies have compared oxytocin or syntometrine with carbetocin. Most of these studies have shown that carbetocin is more effective than oxytocin or syntometrine in prevention of PPH (13-17). However in other studies significant differences between these drugs were not observed (18-20).

Previous studies have reported that the need for additional uterotonic drugs in women who received carbetocin was less than women who received intravenous oxytocin (12, 19); however in other studies, no significant differences were observed between the two groups (16, 20).

The need for additional uterotonic drugs represents the severity of postpartum hemorrhage due to uterine atony, making it an important variable during the outcome measurement.

Recent evidence suggests that carbetocin is safe, similar to oxytocin (13, 14). Moreover, it has fewer adverse effects compared with syntometrine (16, 18). These data indicate that carbetocin can be replaced with syntometrine for the prevention of postpartum hemorrhage.

## 2. Objectives

Based on our knowledge, there is very limited published literature, which compares the use of carbetocin and syntometrine in women who delivered vaginally. Therefore we conducted this randomized trial to compare the efficacy of IM carbetocin with IM syntometrine for the prevention of postpartum hemorrhage.

## 3. Material and Methods

This study was a double-blind, clinical randomized trial that carried was out on 200 women with a singleton pregnancy from March 2011 to June 2011 in the delivery unit of a Shabihkhani Maternity Center in Kashan city in Iran. Exclusion criteria included chronic hypertension, preeclampsia, uterine or cervical rupture, asthma, cardiovascular, renal or liver diseases, grand multiparity, uterine fibroids, and history of PPH.

Patients were randomized to syntometrine (N = 100) or carbetocin groups (N = 100). In the syntometrine group, women received 1 ml of syntometrine (containing 5 units of oxytocin and 0.2 mg ergometrine) and the carbetocin group received 1 ml of carbetocin (containing 100 microgram carbetocin) after placental separation. Randomization was performed using a random number table. Both these drugs were coded and packed before recruitment, and stored at the delivery room. Patients and medical personnel were blinded to the type of drug.

If oxytocin infusion was in progress, it was discontinued. Maternal blood pressure, pulse rate and uterine tonicity were checked immediately after administration of the drugs and repeated 30 and 60 minutes later. Uterine tonicity was scored from zero to 3 which included atony, mild, moderate, and severe tonicity respectively.

The primary outcome measure of this study was the assessment of hemoglobin level on admission to the labour ward and this was compared with hemoglobin level 24 hours after delivery. The secondary outcome measure was the requirement of additional uterotonic medication. In our delivery unit, the criteria for additional uterotonic usage was estimated blood loss of more than 500 ml with or without hypotension or tachycardia and poor uterine tonicity. Women were also evaluated for adverse effects of drugs within 2 hours after delivery.

Eligible women signed the informed consent from and the study protocol, which was approved by the Clinical Research Ethics Committee of Kashan University of Medical Sciences. Also this study was registered in the Iranian registry of clinical trial with this number: 138810212854N2.

### 3.1. Statistical Analysis

All outcome measures, including the need for additional uterotonic agents, uterine tonicity, blood pressure, pulse rate, fall in hemoglobin and drugs side effects were analyzed using Chi-square, Fisher Exact, Student t-tests and logistic regression. The relative risk (RR) and 95% confidence intervals (CI) also were calculated. P value of less than 0.05 (P < 0.05) was considered statistically significant.

### 3.2. Sample Size

Sample size calculations were based on our pilot study, which showed that mean and standard deviation hemoglobin level in carbetocin and syntometrine group was 11.35 ± 1.04 g/dl and 11.79 ± 1.06 g/dl. For 5% level of significance and 80% power it was necessary to recruit 91 women for each trial arm. Considering a dropout rate of 10% the sample size required 100 per group.

## 4. Results

**Figure 1.**
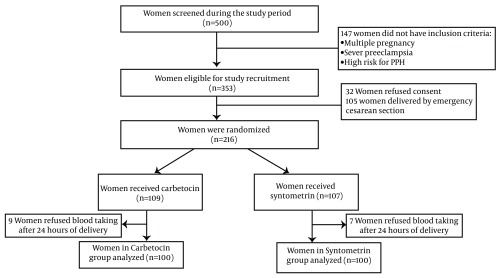
Trial Profile of Recruitment and Randomization to Carbetocin or Syntometrin Groups

Figure 1 is a flow chart of the study design. Women in both groups were comparable in terms of baseline and birth- related characteristics (Table 1). 

**Table 1.** Baseline and Birth-Related Characteristics of the Study Population

Characteristics	Carbetocin (n = 100)	Syntometrine (n = 100)	P Value
**Age (y); Mean (SD)**	25.4 (4.1)	24.8 (4.5)	0.35
**Gestational age (w); Mean (SD)**	38.7 (1.5)	38.8 (1.4)	0.84
**Parity; No. (%)**			0.65
NulliPara	65 (65)	69 (69)	
Multipara	35(35)	31(31)	
**Induction of labour; No. (%)**	75 (75)	76 (76)	0.86
**Episiotomy; No. (%)**	62 (62)	61 (61)	0.88
**Perineal Laceration; No. (%)**	5 (5)	11 (11)	0.19
**Birth weight (g); Mean (SD)**	3208 (152)	3336 (344)	0.45

The primary and secondary outcome measurements of each group are shown in Table 2. The mean fall of hemoglobin concentration after delivery was 0.39 g/dl in the carbetocin group and 1.04 g/dl in the syntometrine group, and the difference was significant. The incidence rate of greater than 10% fall in hemoglobin concentration was 3% and 35% in the carbetocin and syntometrine group, respectively [RR = 11.6, 95% CI (3.7 - 36.7), (P < 0.001)]. Hemoglobin concentration fall of > 20% was seen in 3% of women in the syntometrine group but the difference wasn't significant. 

**Table 2.** Primary and Secondary Outcome Measures of the Study Population

Characteristics	Carbetocin (n = 100)	Syntometrine (n = 100)	P-value
**Hemoglobin at Onset of Labour (g/dl) ** ^**a**^ ****	12.12 ± 1.1	13.02 ± 1.2	0.001
**Hemoglobin at 24 Hours after Delivery (g/dl)** ^**a**^	11.71 ± 1.1	11.98 ± 1.3	0.13
**Mean Fall in Hemoglobin (g/dl)** ^** a**^	0.41 ± 0.36	1.04 ± 0.78	< 0.001
**Percent Fall of Hemoglobin **			
> 20%	0 (0)	3 (3)	0.08
> 10%	3 (3)	35 (35)	< 0.001
**Need for Additional Uterotonic**	1 (1)	11 (11)	0.002
**Uterine Tonicity Immediately after Drug Administration** ^**a**^	2.11 ± 0.54	1.29 ± 0.47	< 0.001
**Uterine Tonicity 30 Minutes after Drug Administration** ^**a**^	2.01 ± 0.59	1.68 ± 0.46	< 0.001
**Uterine Tonicity 60 Minutes after Drug Administration** ^**a**^	2.04 ± 0.63	1.94 ± 0.23	0.14
**Mean Systolic Blood Pressure Immediately after Drug Administration** ^**a**^	112 ± 91	120 ± 73	< 0.001
**Mean Systolic Blood Pressure 30 Minutes after Drug Administration** ^**a**^	109 ± 78	115 ± 77	< 0.001
**Mean Systolic Blood Pressure 60 Minutes after Drug Administration** ^**a**^	111 ± 69	113 ± 85	0.03
**Mean Diastolic Blood Pressure Immediately after Drug Administration** ^**a**^	71 ± 9	73 ± 8	0.05
**Mean Diastolic Blood Pressure 30 Minutes after Drug Administration** ^**a**^	70 ± 8	71 ± 7	0.57
**Mean Diastolic Blood Pressure 60 Minutes after Drug Administration** ^**a**^	71 ± 7	71 ± 7	0.88

^a^ Data are presented as Mean ± SD

Eleven percent of women in the syntometrine group required additional uterotonic agents but 1% of women in the carbetocin group required the use of additional uterotonic agents and there were signiﬁcant difference between the two groups [RR = 11, 95% CI (1.44 - 83.6),(P = 0.002)].

The mean uterine tonicity values at different intervals after drug administration also are shown in Table 2. There was a difference between the carbetocin group and syntometrine group in terms of uterine tonicity immediately and 30 minutes after drug injection (P < 0.001). 

The mean blood pressure values at different intervals after drug injection in the two groups are shown in Table 2. There were significant differences between the two groups in terms of systolic blood pressure immediately, 30 and 60 minutes after drugs injection (P < 0.001) but not in the diastolic blood pressure at the same times. None of the women in this study had hypertension (blood pressure > 140 / 90 mmHg) at 0, 30 and 60 min after drug injection 

Adverse effects of these drugs are presented in Table 3. Women in the carbetocin group experienced fewer symptoms such as nausea, abdominal pain and chill but the difference wasn't significant. 

**Table 3.** Adverse Effects of Two Drugs in the Study Population

Characteristics	Carbetocin (n = 100)	Syntometrine (n = 100)	P-value
**Nausea**	2%	3%	0.65
**Vomiting**	1%	0%	0.5
**Chill**	0%	1%	0.5
**Abdominal Pain**	1%	0%	0.5
**Hypotension (BP < 90 / 60 mmHg)**	0%	2%	0.49
**Tachycardia (Pulse ≥ 100 Beats per Minute) Immediately After Delivery**	13%	5%	0.04

Multivariate analysis with regression logistic showed that baseline factors such as age parity, gestational age, induction of labor, episiotomy, perineal laceration, birth weight and hemoglobin level before delivery was not associated with fall of hemoglobin level, but the difference between the two drugs was significant.

## 5. Discussion

This was the ﬁrst randomized controlled trial assessing the effects of carbetocin in Iran. Several randomized trials have compared oxytocin or syntometrine with carbetocin. Most of these trials have found carbetocin to be better than oxytocin or syntometrine in prevention of PPH (13-17), but in three studies there weren't any significant differences between these drugs (18-20).

Our study showed that in the syntometrine group, mean fall of hemoglobin concentration after delivery and fall in hemoglobin level more than 10% was higher than the carbetocin group, and their difference was significant. This shows that IM carbetocin is more effective than syntometrine in prevention of postpartum hemorrhage. Several studies have shown that fall in hemoglobin level in the syntometrine group was higher than the carbetocin group (15-17). These findings are compatible with our results and indicate the better effect of carbetocin than syntometrin. However Leung and Boucher studies indicate no difference in the fall of hemoglobin concentration between the two groups (17, 18). Our findings are different from these researches. There may be several reasons for this, most importantly the difference in the eligibility criteria (such as type of delivery or high risk of PPH), type of drug (such as IV oxytocin), syntometrin dosage and timing of the intervention.

Our study showed that carbetocin is more effective than syntometrine in terms of the need for additional uterotonic agents and the difference was statistically signiﬁcant. One of the important indicators of postpartum bleeding is the need for additional uterotonic agents. Su et al. have shown that the need for additional uterotonic agents was 13.5% in the carbetocin group and 16.8% in the syntometrine group but the difference wasn't signiﬁcant (20). In addition, the same results have been shown by several other studies (16, 18, 19).

Our results indicated that there was a difference between the carbetocin group and syntometrine group in terms of uterine tonicity after delivery. This issue revealed the better effect of carbetocin than syntometrin on uterine tonicity during 24 hours after delivery. Dansereau et al. have found that carbetocin is more effective than oxytocin in maintaining uterine tone (14). One study showed that injection of carbetocin after delivery at a single dose can cause a uterine contraction in less than 2 minutes and will continue for two hours (11). However Ngan et al. showed that uterine tone in the two groups was similar (15).

Based on the findings of this study there was a significant difference between the two groups in terms of systolic blood pressure at 0, 30 and 60 minutes after drug administration but this difference wasn't significant in terms of diastolic blood pressure. None of the women in this study had blood pressures greater than 140/90 mmHg. In one study, significant increases in systolic and diastolic blood pressure at 30 and 60 min after delivery were seen in the syntometrine group (16). Although in the Ngan et al. study, blood pressure was similar between the two groups (15) and in the Leung et al. research, the rate of high blood pressure in the carbetocin group was considerably lower than the syntometrine group (18).

Our findings show that mean and standard deviation of heart rate between the two groups were statistically signiﬁcant only immediately after drug administration. Also, the incidence rate of tachycardia (PR > 100BPM) in the carbetocin group was 13%, in contrast to 5% in the syntometrine group (P = 0.04). A previous study has shown that carbetocin can cause maternal tachycardia (9, 11). In the Leung study 21% of women who received carbetocin had tachycardia, which was significantly greater than women who received syntometrine (18). This finding is virtually the same as our results. However in two studies the pulse rate was similar between the groups (15-17).

In this study the incidence of many adverse effects, such as nausea, vomiting, chill, abdominal pain and hypotension was low and similar in the two groups. In the Askar et al. study, women in the carbetocin group had lower incidence of nausea and vomiting, and the difference between the two groups was statistically signiﬁcant. However, the incidence of other adverse effects such as ﬂushing, headache, and abdominal pain were low and similar for both groups (16). Also Leung et al. study has shown that the incidence of nausea and vomiting in the carbetocin group is lower than the syntometrine group (18).

The difference between our findings and other studies in terms of adverse effects and additional uterotonic requirements could be due to differences in syntometrine dosage or type of uterotonic drugs that was compared with carbetocin.

Our results showed that carbetocin is more effective than syntometrin in prevention of postpartum hemorrhage. The need for additional uterotonic agents was lower in women who received carbetocin; moreover its adverse effects were low. Therefore it can be concluded that IM carbetocin is a good alternative of IM syntometrine in low-risk women.
